# A deep learning approach for ovarian cancer detection and classification based on fuzzy deep learning

**DOI:** 10.1038/s41598-024-75830-2

**Published:** 2024-11-02

**Authors:** Eman I. Abd El-Latif, Mohamed El-dosuky, Ashraf Darwish, Aboul Ella Hassanien

**Affiliations:** 1 Faculty of Science, Benha University, Benha, Egypt; 2 Computer Science Department, Arab East Colleges, Riyadh, Saudi Arabia; 3 Computer Science Department, Faculty of Computers and Information, Mansoura University, Mansoura, Egypt; 4 Faculty of Science, Helwan University, Cairo, Egypt; 5 Faculty of Computers and Artificial Intelligence, Cairo University, Cairo, Egypt; 6 Scientific Research school of Egypt (SRSEG), Cairo, Egypt

**Keywords:** Ovarian cancer, ResNet-50, Recursive feature elimination (RFE), Fuzzy logic, Deep learning, Wang–Mendel, Cancer, Medical research

## Abstract

Different oncologists make their own decisions about the detection and classification of the type of ovarian cancer from histopathological whole slide images. However, it is necessary to have an automated system that is more accurate and standardized for decision-making, which is essential for early detection of ovarian cancer. To help doctors, an automated detection and classification of ovarian cancer system is proposed. This model starts by extracting the main features from the histopathology images based on the ResNet-50 model to detect and classify the cancer. Then, recursive feature elimination based on a decision tree is introduced to remove unnecessary features extracted during the feature extraction process. Adam optimizers were implemented to optimize the network’s weights during training data. Finally, the advantages of combining deep learning and fuzzy logic are combined to classify the images of ovarian cancer. The dataset consists of 288 hematoxylin and eosin (H&E) stained whole slides with clinical information from 78 patients. H&E-stained Whole Slide Images (WSIs), including 162 effective and 126 invalid WSIs were obtained from different tissue blocks of post-treatment specimens. Experimental results can diagnose ovarian cancer with a potential accuracy of 98.99%, sensitivity of 99%, specificity of 98.96%, and F1-score of 98.99%. The results show promising results indicating the potential of using fuzzy deep-learning classifiers for predicting ovarian cancer.

## Introduction

The 8th most common cancer in women and the 18th most frequent cancer overall is the ovarian cancer^1^. Ovarian cancer is the deadliest of gynecologic tumors and it represents 2.5% of cancers in women. Ovarian cancer occurs when the genetic material (DNA) undergoes changes and the cells in the ovaries increase rapidly and can attack the solid body tissue^2^. Additionally, ovarian cancer can be happened by inherited genetic changes such as BRCA1 and BRCA2^3^. Ovarian cancer comes in a variety of types. Epithelial cancer is the most prevalent kind. It starts in the ovary’s protective cells^4^. In 2020, over 207,252 new deaths globally are reported and close to 313,959 are diagnosed with ovarian cancer^5^. Ovarian cancer rates are the highest in Brunei, followed by Samoa and the highest death rate for Samoa in 2020^6^. According to Ovarian Cancer Statistics, in 2023, there are 21,410 new cases of ovarian cancer in the United States and 13,770 deaths from the disease^7^.

After years of research, there are still no trustworthy diagnostic methods that can identify cancer early on and be used for screening. Furthermore, there may be no early warning signs or symptoms of ovarian cancer. Therefore, ovarian cancer is diagnosed in the advanced stages, when the tumors have spread. Contingent upon the phases of the infection, the treatment approach might comprise a medical procedure, chemicals and radiation. Therefore, a disease prognosis is very important, especially for cancer and other cancers that are malignant^8^. So, the main component that assists clinicians with settling on proper medicines. Patients’ anxiety can be reduced, and their knowledge of treatment options improved by survival prediction.

The exploration and application of artificial intelligence (AI) in medicine in recent years have altered our understanding of traditional medical techniques in connection with the development of the science and technology^9,10^. AI constructs information models to work out, make expectations on tremendous clinical information and solves the medical issues that are hidden behind the data. Currently, artificial intelligence can introduce effective alternatives to diagnose various types of cancer using machine and deep learning models^11,12^. Deep learning for medical applications will improve productivity, decrease manual input and provide radiologists with important information that are invisible to the human eye, and enable more accurate than current techniques. Deep learning automatically identifies significant characteristics that can identify extremely subtle symptoms from histopathology images.

This paper proposed an intelligent and automatic model that detect and classify ovarian cancer based on histopathology images. The proposed model addresses the restrictions of traditional techniques by leveraging the power of deep learning and recursive feature elimination, which allows for more efficient and accurate prediction.

The proposed model starts by increasing the number of datasets through the augmentation process using scaling, vertical flipping, and rotation to avoid over-fitting and enhance the size and quality of training datasets. Then, with the help of the ResNet50 model, a simple CNN with 50 deep layers, the features are extracted from histopathology images. Additionally, recursive feature elimination is a vital technique applied to enhance the effectiveness of images classification by removing unnecessary or useless features. Finally, fuzzy deep learning classifier is performed to train the model and classifying the dataset. A benchmark dataset consists of 288 hematoxylin and eosin (H&E) stained whole slides with clinical information from 78 patients. H&E-stained Whole Slide Images (WSIs), including 162 effective and 126 invalid WSIs were obtained from different tissue blocks of post-treatment specimens utilized to train and validate the proposed model to predict ovarian cancer patients. The following briefly describes the primary contributions of this paper:

- The proposed model uses the data augmentation approach to overcome the issue of limited data.
- ResNet50, a simple CNN with 50 deep layers, is utilized to extract the main features from the histopathology images
- Recursive feature elimination based on a decision tree is introduced to remove unnecessary features extracted during the feature extraction process.
- A novel approach to investigate the advantages of merging fuzzy logic with deep learning for classification of the ovarian cancer images and the Wang-Mendel method is applied for generating the rules.
- The developed model has a potential accuracy reaching 98.99% using a fuzzy deep learning algorithm to identify ovarian cancer using histopathology images.

The remaining sections of this paper are structured as follows. Section 2 reviews the related work. Section 3 focuses primarily on the background. The proposed model is presented in Section 5. Additionally, section 6 presents the experimental results and the evaluation metrics of the proposed model. Section 7 concludes the work and offers recommendations for further research.

## Related work

The prognosis for cancer has been predicted using a variety of studies^13,14^. Some of these studies focused on statistical techniques and deep learning to forecast the prognosis of ovarian cancer patients as shown in Table 1.

**Table 1:** Summary of ovarian cancer classification approaches

Ref.	Methodology	Dataset	Performance evaluation
^15^	Statistical tests such as skewness and Pearson correlation coefficient are used for classification and regression: KNN, SVM, DT, RF, AdaBoost, and XGBoost	Surveillance, epidemiology, and end results (SEER)	For classification: RF achieves the best performance with 88.72% accuracy and 82.38%AUC For regression XGBoost with 20.61% RMSE and 0.4667 R^2^
^16^	Convolutional neural network model with a convolutional autoencoder is performed and classify five diseases which are: normal, cystadenoma, mature cystic teratoma, endometrioma, and malignancy.	1613 ultrasound images	For 2 classes: 97.2% accuracy, 97.2% sensitivity, 0.9936 AUC For five classes: 90.12% accuracy, 86.67% sensitivity, 94.06% AUC
^17^	-There are two experiments and for each experiment, three methods are applied. -The first experiment was divided into two distinct subsets (65% training and 35% testing) -5-fold cross validation was carried out for the second experiment.	There are two datasets: – The first data contains 288 hematoxylin and eosin slides – The OORE tissue microarray (TMA) dataset contains 175 tissues	Method 3 achieves the highest accuracy 88.2% in the first dataset using 5-fold cross validation. -Method 3 achieves the highest accuracy 77.5 % in the TMA dataset
^18^	DenseNet with 121 layers and gradient descent using 0.001 learning rate, 0.9 momentum, 32 minibatches and 0·0001weight decay.	– internal dataset (575,930 ultrasound images) – internal validation dataset (8416 ultrasound images) – External validation dataset2 (6510 images)	AUC values are 0.911, 0.870, and 0.831 in the internal dataset, external validation first dataset, and external validation second dataset, respectively.
^19^	CNN with 2 convolutional layers, ReLU activation and Adam optimizer	11,040 histopathological images	94.43% accuracy, 95.02 sensitivity, 93.16 specificity

In last years, many researchers have focused on enhancing cancer detection results using histopathological images and diagnostic tests such as CA-125 and HE-4. But these diagnostic tests are slow and have restricted exactness in detecting ovarian cancer in the beginning phase^20^. In addition, imaging techniques such as resonance imaging tests (MRIs), computed tomography scans (CT), and Ultrasound can be time-consuming and costly.

Many algorithms have been offered to extract features using machine learning techniques and then categorize ovarian tumors. A few procedures include wavelet coefficients, and multi-scaling for feature extraction while using SVM and neural networks for classification.

El-Bendary et al.^21^ presented an algorithm based on boosting and ensemble SVM for ovarian cancer classification. In^22^, the authors used the T-POT optimization technique and KNN classifier for detecting ovarian cancer. They classified normal, malignant, borderline, and benign cases by examining S-HG images. Jamshid et al.^15^ applied skewness and the Pearson correlation coefficient to calculate the significance of the features from the SEER database. Then, six machine learning models are employed for classification, which are KNN, SVM, DT, RF, AdaBoost, and XGBoost. For prediction, interpretable methods and Shapley additive explanations are applied to extract the significance of each feature in the prediction.

In recent years, various techniques have utilized deep learning for early diagnosis the ovarian cancer. The studies demonstrated the potential of deep learning for subtyping and detection of ovarian cancer utilizing a variety of data, counting gene expression, histopathological and ultrasound images. These techniques have demonstrated potential improvements in accuracy and molecular subtype identification.

Yuyeon, et al.^16^ developed the CNN-CAE model that categorizes ovarian tumors into five groups and eliminates redundant information from ultrasound images. The CNN-CAE model demonstrated 96.0% accuracy in categorizing ovarian tumors as benign, and 86.85% accuracy in classifying the various forms of ovarian tumors.

Ching-Wei et al.^17^ build a model to accurately predict ovarian cancer patients from histopathological images using two datasets. Two experiments are performed. For the first experiment, the dataset is divided into two distinct sections (65% training and 35% testing), and 5-fold cross-validation is carried out for the second experiment.

Yue et al.^18^ performed a dense convolutional network with 121 layers to distinguish between benign and malignant ovarian cancer from ultrasound images. In this research, three datasets are used for evaluating the model. Firstly, the training dataset contains 575,930 ultrasound images from 105,532 persons (3755 has ovarian cancer and 101 777 do not). The second dataset contains 8416 ultrasound images are collected from 868 cases (266 ovarian cancer/ 602 not cancer) to generate the internal validation dataset. Lastly, external validation dataset 2 included 6510 images from 889 cases, 1253 of which were benign and 5257 of cancerous ovarian tissue.

In^19^, a histopathology image dataset is augmented and then split into training and validation subsets for training using CNN The model archives 94% accuracy rate, correctly identifying 95.12% of cancer cases and 93.02% of healthy cases.

## Preliminary

In this section, we will cover the fundamental ideas and algorithms presented in the proposed model including ResNet, Recursive Feature Elimination (RFE), fuzzy layer, and Generated Rules based on Wang-Mendel.

### ResNet-50

Residual Network is referred to as ResNet. It is a cutting-edge neural network that He et al. first demonstrated^23^. Residual Neural Network (ResNet) builds a network by stacking residual blocks on top of each other. The ResNet model has an advantage over other architectural models in that the performance does not suffer as the design becomes deeper and the network training is better. The vanishing gradient problem was the inspiration behind the creation of the Residual Blocks concept. This network uses a technique called skip connections. By excluding connections on two to three levels that include ReLU and batch normalization between the architectures, the ResNet model is implemented. The residual block on ResNet can be calculated as follows:

1 $$y=F\left(x, W + x\right)$$

Where $x$ is input layer; $y$ is output layer; $W$ is the weight and $F$ function is denoted by the residual map.

ResNet-50 contains 48 convolutional layers, 1 Max pooling layer, and 1 average pooling layer. The first layer of the ResNet architecture consists of a max-pooling with a size of $3\times 3$ and a convolution of $7\times 7$ with a stride of 227. If the data are not normalized during training, the network may have problems, which would make training much more challenging and slow down its learning rate. Instead of using the entire data set, batch normalize is performed in smaller batches. It speeds up preparing and making learning easier. Batch normalization is accomplished by fixing the means ${\upmu }_{\text{B}}$ and variance ${\upsigma }_{\text{B}}^{2}$ of each input layer during the normalization step and it calculates using the following equations:

2 $$\mu _{B} = \frac{1}{m}\sum\limits_{{i = 1}}^{m} {x_{i} }$$

3 $$\sigma _{B}^{2} = \frac{1}{m}\sum\nolimits_{{i = 1}}^{m} {(x_{i} - \mu _{B} )^{2} }$$

Where $B$ denotes the mini batch of the size m of the whole training set.

The Dropout layer is another critical component of ResNet. The Dropout layer acts as a mask, lowering some neurons’ contributions to the following layer while maintaining all other neurons operational. Upon applying a Dropout layer to an input vector, certain characteristics of the vector are diminished; however, when we apply it to a hidden layer, some hidden neurons are eliminated. Dropout layers are critical in ResNet training because they prevent the training data from being overfit. Finally, the classification step is carried out by the CNN’s fully connected layer. Because every neuron in the next layer is related to every neuron in the preceding layer, the choice is genuinely dependent on the entire image in the FC layer. ResNet has been used in a wide range of applications, including object identification^24^, medical images classification^25^ and semantic segmentation^26^. Medical imaging applications like CT and MRI scans have used ResNet to classify and segment various structures in the images^27^.

### Recursive feature elimination: feature selection approach

Recursive feature elimination (RFE) is a feature selection technique for locating the important characteristics in a dataset^28^. The aim of feature selection is to find the best features that can simultaneously achieve dimension reduction and accurate prediction. A technique for iteratively eliminating less important features while concentrating on those that improve predicted accuracy is called recursive feature elimination, or RFE.

Until a desired feature subset is found, RFE assesses the relevance of each feature, eliminates the least important, and rebuilds the model. RFE takes feature interactions into account, unlike filtering and wrapper techniques. Although it could be computationally taxing, it provides reliable performance in complicated datasets.

To create classifiers that are more effective, REF is frequently used with other classification techniques (such as SVM, DT, etc.). Decision tree (DT) is one of the classifiers most frequently used in recursive elimination. With every leaf node indicating the outcome and each internal node representing an attribute, DT is a tree structure that simulates a flowchart^29^. A decision tree’s root node is found at the very top. It gains the ability to divide data according to an attribute’s value. Although there are many approaches to choose the optimal attribute at each node, the entropy and information gain methods are the two that are most frequently used as a splitting criterion in decision tree models. Entropy derived from information theory that evaluates the impurity of values in data. Information gain is the difference in entropy between before and after an attribute split. Entropy is denoted with the following formula:

4 $$E = \sum\limits_{{i = 1}}^{n} {p_{i} log_{2} \left( {p_{i} } \right)}$$

Where the probability of choosing an example from class $i$ at random is the value of ${p}_{i}$.

**Fig. 1:**
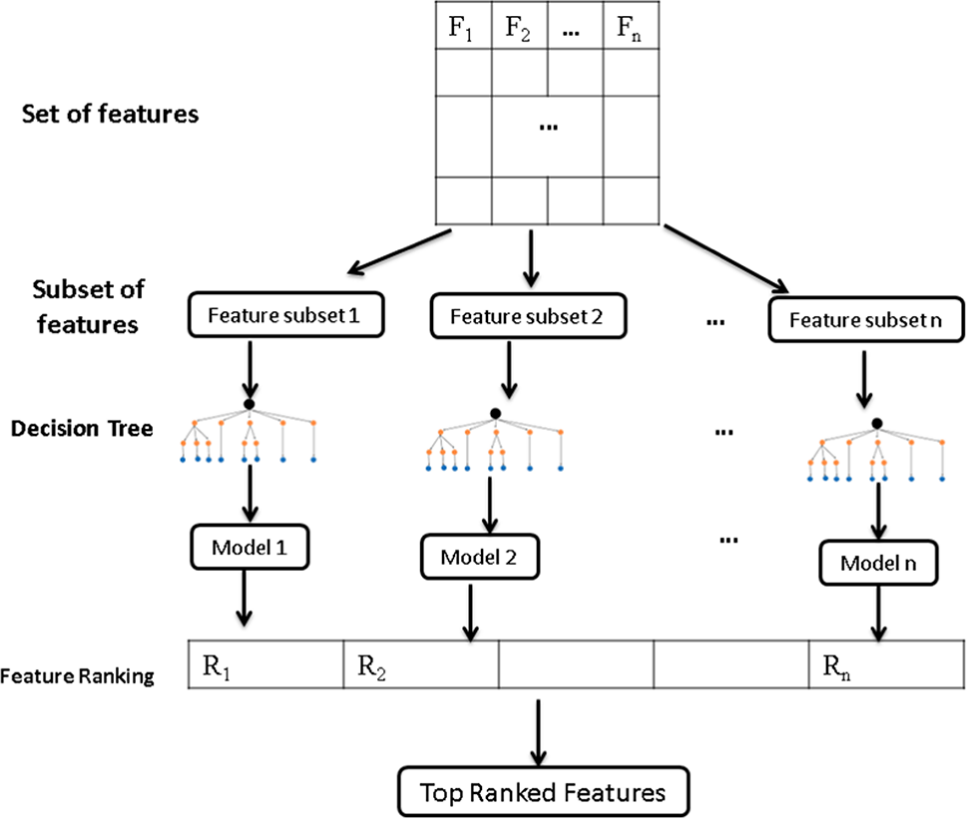
Recursive feature elimination with decision tree

In the first step in the recursive feature elimination, the features split into sub-features and then create a model for each sub-feature using decision tree^30^. The model is then rebuilt, significance feature is calculated, and the least significant features are discarded as shown in Fig. 1.

### Fuzzy layer

Lotfi Zadeh invented fuzzy logic to represent the type of data utilized in information processing and to derive the essential logical rules for this type of set. Fuzzy logic is a variable processing method that permits numerous truth values to be processed by the same variable. Fuzzy logic and machine learning are frequently confused, although they are not the same thing. Machine learning refers to computing systems that tackle complicated problems by iteratively changing algorithms to emulate the thinking of humans. Although fuzzy logic is a set of rules and functions that may work on inaccurate data sets, the algorithms must still be programmed by humans^31^. Fuzzy systems based on fuzzy IF-THEN rules have been created and used in a wide range of fields in recent decades. In fuzzy systems, fuzzy rules are crucial since their effectiveness determines the system’s capabilities. Extraction of fuzzy rules from data is the fundamental to developing a fuzzy system. There are several techniques for extracting fuzzy rules, including heuristic techniques, genetic algorithm and fuzzy clustering techniques. However, these techniques either need repetitive learning or costly time due to complicated mechanics. The MFs are the fundamental units of fuzzy set theory. Member functions (MFs) come in a variety of forms, including triangular, trapezoidal and Gaussian. Gaussian function has less parameters and better partial derivatives for parameters. The Gaussian membership function is calculated using the following equation:

5 $$\mu _{{ij}} = {\text{exp}}\left( {\frac{{(x_{{sj}} - c_{{ij}} )^{2} }}{{2\sigma _{{ij}} }}} \right)$$

Where ${\mu }_{ij}$ is the membership function at rule $i$ and feature , ${x}_{sj}$ represent $s$ sample in feature $j$ and ${\sigma }_{ij}$ and ${c}_{ij}$ are the center and the width of Gaussian function, respectively.

### Generated rules based on Wang–-Mendel

**Fig. 2:**
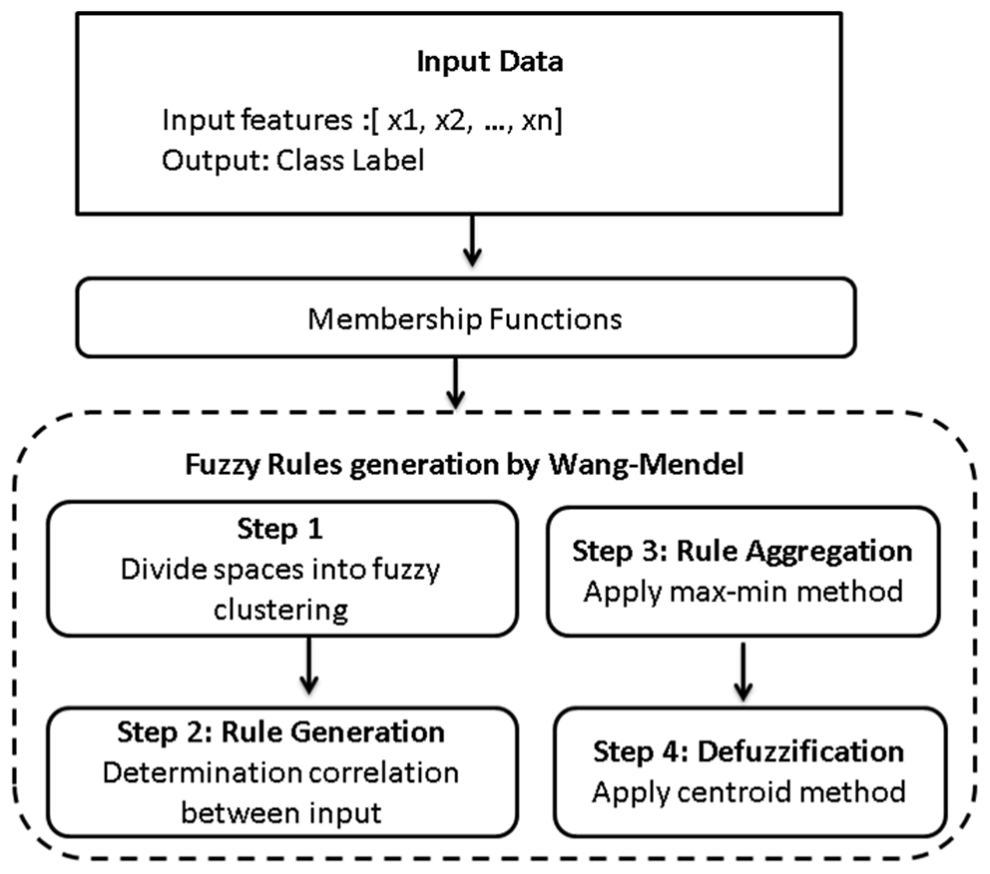
Wang–-Mendel steps for fuzzy generation rules

The Wang–-Mendel (WM) approach, developed by Wang and Mendel^32,33^, effectively produces fuzzy rules from input data. The performance of fuzzy representation as a whole is greatly influenced by the membership functions. The Wang-Mendel method has five steps to produce rules. They are fuzzy clustering, fuzzy set creation, rule generation, rule aggregation, and defuzzification as shown in Fig. 2. The Fuzzy clustering can be written as:

6 $$J_{m} = \sum\nolimits_{{i = 1}}^{N} {\sum\nolimits_{{j = 1}}^{C} {w_{{ij}}^{n} \left\| {x_{i} - \mu _{j} } \right\|} }$$

Where $N$ is the number of data points, $C$ is the number of clusters, ${x}_{i}$ is the ${i}^{th}$ data point,${\mu }_{j}$ is the centroid of the ${j}^{th}$ cluster, ${w}_{ij}$ is the degree of membership of the ${i}^{th}$ data point in the ${j}^{th}$ cluster, and m is a weighting exponent that determines the fuzziness of the clustering. The membership function for a fuzzy set can be written as:

7 $$\mu _{A} \left( x \right) = \frac{1}{{1 + \left( {\frac{{x - c}}{a}} \right)^{{2b}} }}$$

Where $c$ is the center of the fuzzy set, $a$ is the spread (width) of the fuzzy set, and $b$ is a parameter that controls the shape of the membership function.

The max-min method for rule aggregation can be written as:

8 $$\mu _{B} \left( y \right) = {\text{max}}(min\,\mu _{A} \left( {x_{i} } \right),\mu _{B} ^{\prime } (y)$$

${\mu }_{A}\left({x}_{i}\right)$ is the degree of membership of ${x}_{i}$ in the ${i}^{th}$ antecedent fuzzy set,${{\mu }_{B}}{\prime}(y$ ) is the degree of membership of $y$ in the output fuzzy set before aggregation, and ${\mu }_{B}\left(y\right)$ is the degree of membership of $y$ in the output fuzzy set after aggregation.

The centroid method for defuzzification can be written as:

9 $${\text{y}}_{{{\text{crisp}}}} = \frac{{\int_{{ - \infty }}^{\infty } {{\text{y}} \upmu _{{\text{B}}} ({\text{y}}){\text{dy}}} }}{{\upmu _{{\text{B}}} ({\text{y}}){\text{dy}}}}$$

where ${y}_{crisp}$ is the crisp output value, $y$ is the output variable, and ${\mu }_{B}(y)$ is the degree of membership of $y$ in the output fuzzy set after aggregation.

## Data characteristics

**Fig. 3:**
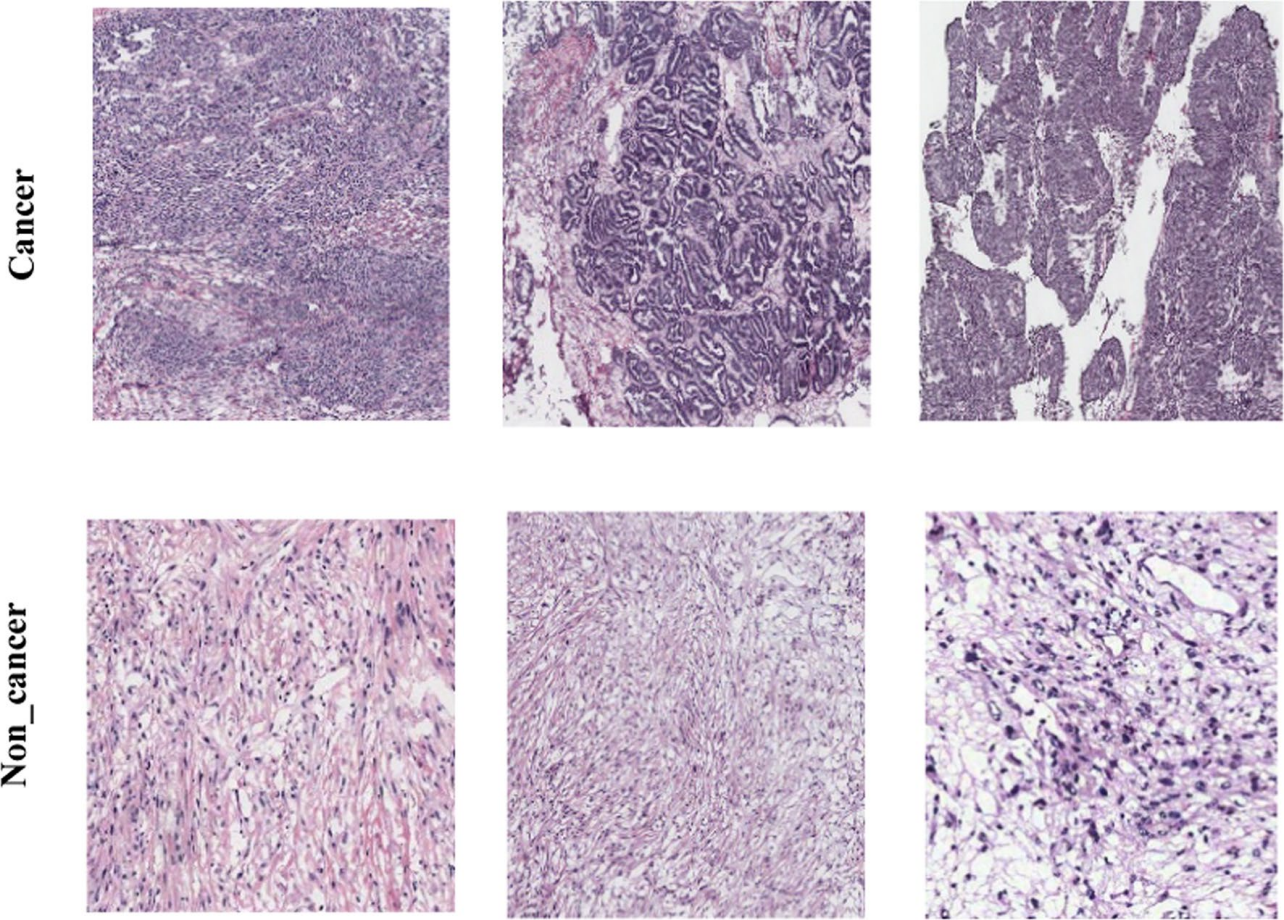
Samples of the cancer and noncancer

The dataset comprises of hematoxylin and eosin (H&E) stained entire slides that collected from 78 patients^34^. The slides are gathered from the tissue bank of the Tri-Administration General Emergency clinic and the Public Guard Clinical Center, Taiwan. With a 20-objective lens on a digital slide scanner (Leica AT2), dataset was collected. In our dataset, we have 481 samples for cancer class and 506 noncancer class. The top row in Fig. 3 represents the cancer sample, and the second row the noncancer sample.

## The proposed ovarian *cancer* model

**Fig. 4:**
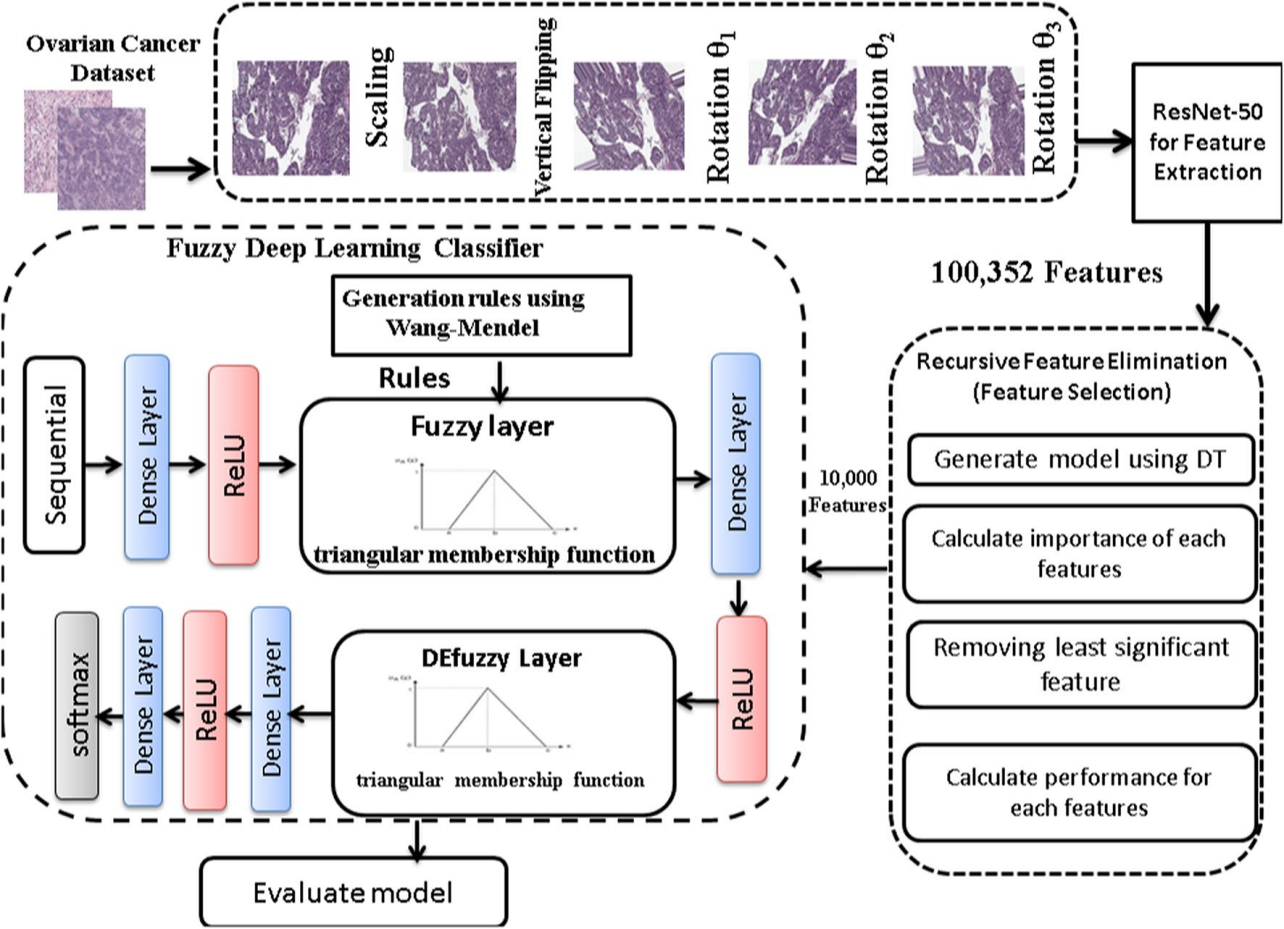
The general proposed architecture of the model

The general consensus in computer-diagnosis analysis schemes is that rather than attempting to make a computer operate like a diagnostician, the method should provide physicians with useful computer-generated information for decision assistance. Firstly, the number of the benchmark images of ovarian cancer are increased through the augmentation process to avoid over-fitting and increase the size of training datasets. Then, ResNet50 is a 50-deep-layer convolutional neural network that extracts features from images. To improve the algorithm’s performance in the high-dimensional feature, feature selection technique was applied. Recursive feature elimination is performed to reduce the dimensionality of the training data by choosing the most crucial features. Using fuzzy and deep learning techniques, the classification stage is completed by separating cancer and non-cancer from histopathology images. The general architecture of the proposed model is illustrated in Fig. 4. The architecture is composed by four fundamental building processes: data augmentation, feature extraction, feature selection, and deep fuzzy -based classification. In the following subsections, we will discuss in detail each process.

### Phase 1: data augmentation

The images in the dataset are augmented using Algorithm 1 through the use of flipping, scaling, and rotation operations. Through this process, the model’s training is improved, making it more robust and less prone to overfitting. The first step in this augmentation procedure is to initialise the empty list Result, which hold the original and modified images. The algorithm first reads each image in the dataset, then appends the original image to the Result list. Next, it creates a new scaled version of the image, which is included to the Result, by scaling it by 20%. Subsequently, the algorithm produces three random angles which are within a specified range of 50 degrees. It then rotates the image by each of these angles to generate three unique images with different rotations. After that, each of these rotated images is included in the Result list. By the end of the algorithm, the original and augmented images are included in the Result list.

**Algorithm 1:**
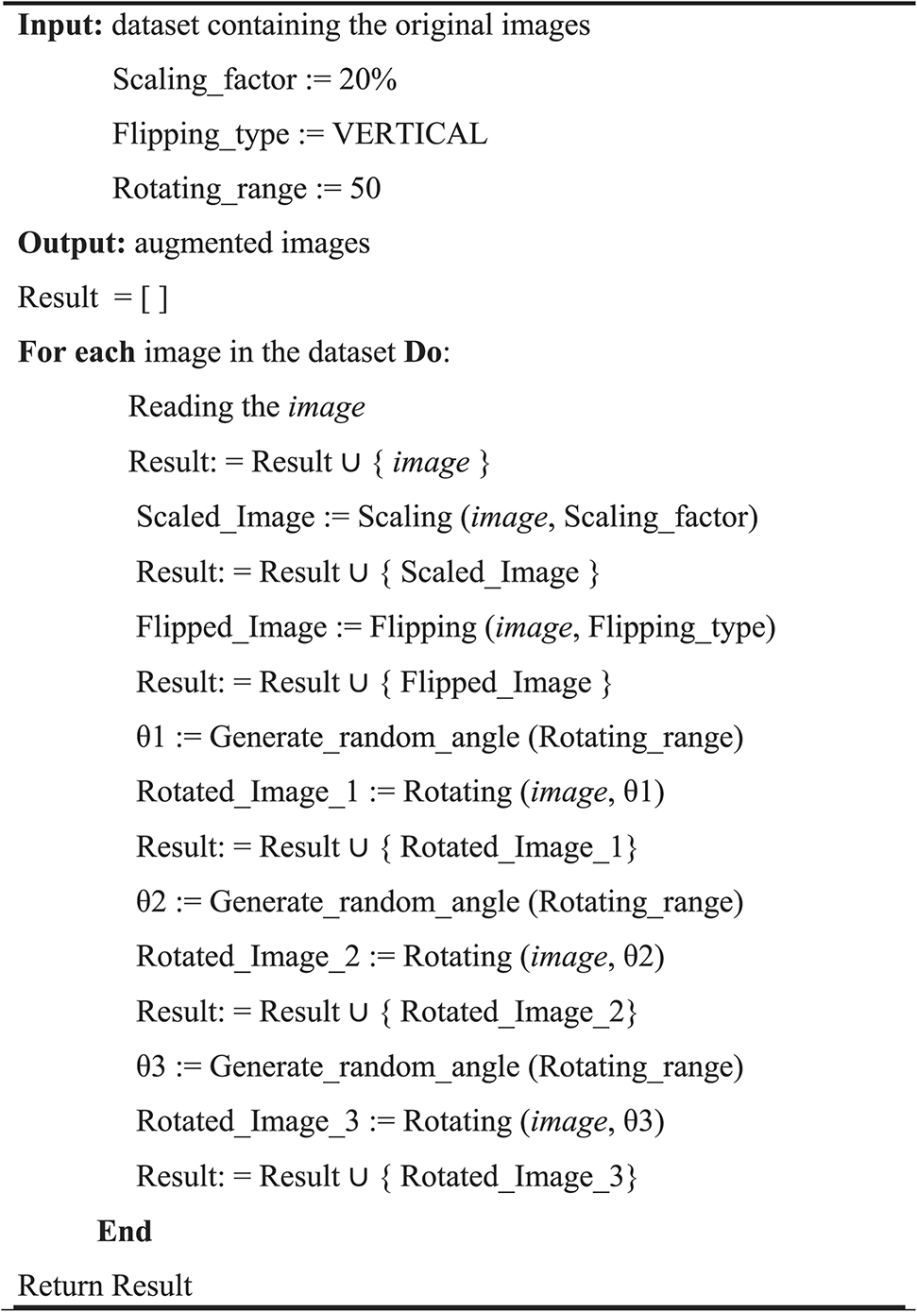
Image augmentation

### Phase 2: feature extraction using ResNet-50

Algorithm 2 starts with a number of augmented images as input and outputs a set of features that have been extracted. Each image and their labels are used by the algorithm to create an empty list called batchImages, which holds the images that will be sent to ResNet50 for feature extraction. The algorithm resizes each image to $224 \times 224$ pixels and removes the average pixel intensity from the dataset to normalize the image. Following processing, these images are added to batchImages. Once the images are resized, it is transmitted to the ResNet50 model. ResNet50 creates features for these images, which are then reduced to a size of $7\times 7\times 2048$. In the end, the algorithm combines each extracted characteristic with the matching class label in a file.

**Algorithm 2:**
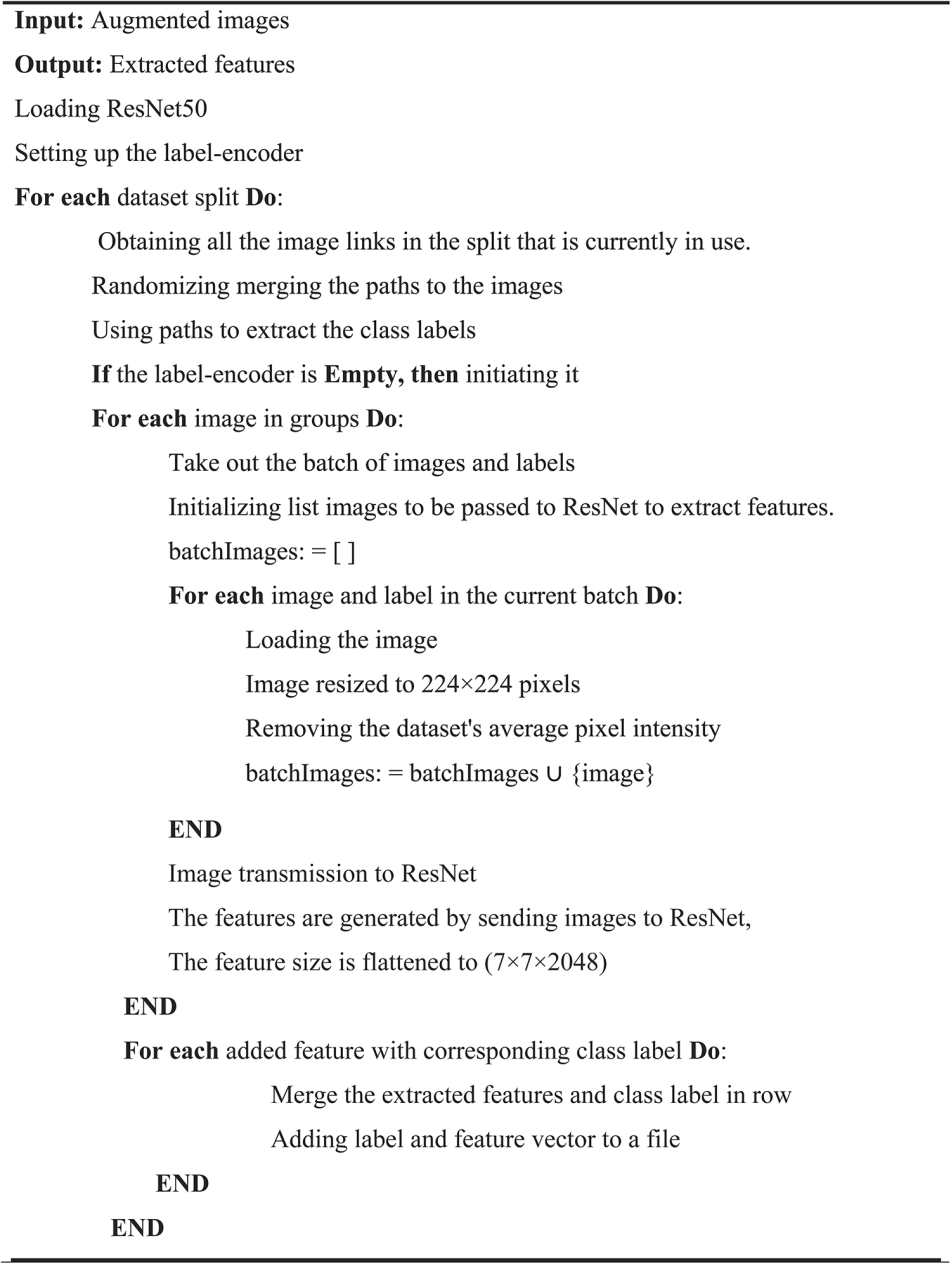
ResNet50 feature extraction algorithm

### Phase 3: feature selection with recursive feature elimination (RFE)

Algorithm 3 explains the feature ranking method called Recursive Feature Elimination (RFE), which is based on a Decision Tree. The algorithm’s goal is to provide a ranked list of features according to their importance. It starts by accepting a set of features F_i_ and a Decision Tree as inputs. To enable a comprehensive examination, the features are subsequently separated into *N*. Train the Decision Tree model with the remaining features for each of the N folds. Then, evaluate the trained model with the fold’s features to determine its accuracy score. This procedure is carried out once more for each of the N folds, after which the average accuracy over all folds is computed. The features are ranked according to the computed accuracies. The feature that has the least impact on accuracy, or the least significance, is found and eliminated. After that, the Decision Tree model is retrained using the new set of features. This procedure is performed recursively until the required number of features is obtained or until another stopping criterion is satisfied. In conclusion, Algorithm 3 enhances the model’s performance by concentrating on the most significant characteristics through the iterative removal of the least important features utilizing recursive feature elimination with a Decision Tree. This method removes unnecessary or uninformative characteristics to assist create a more accurate and efficient model.

**Algorithm 3:**
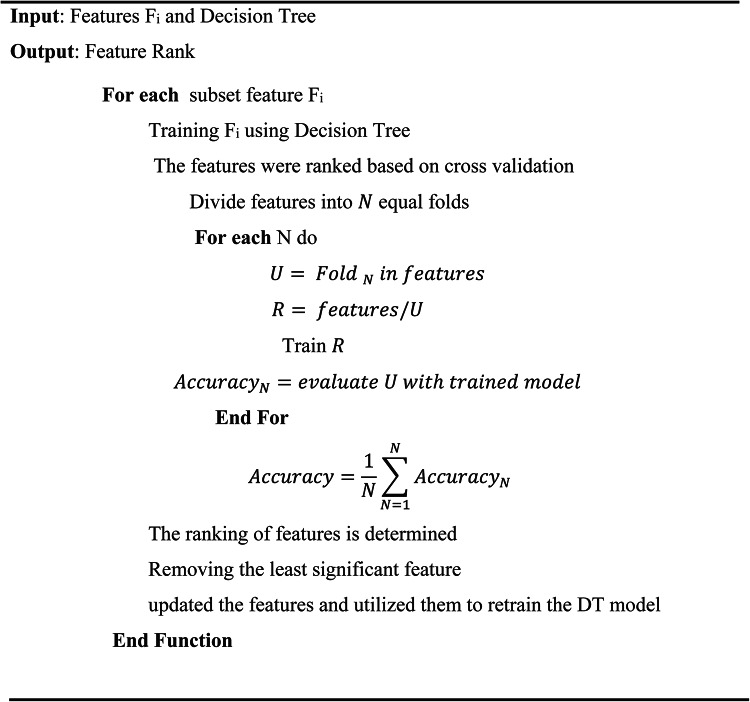
Recursive feature elimination based ondecision tree

### Phase 4: fuzzy deep learning classifier

**Fig. 5:**
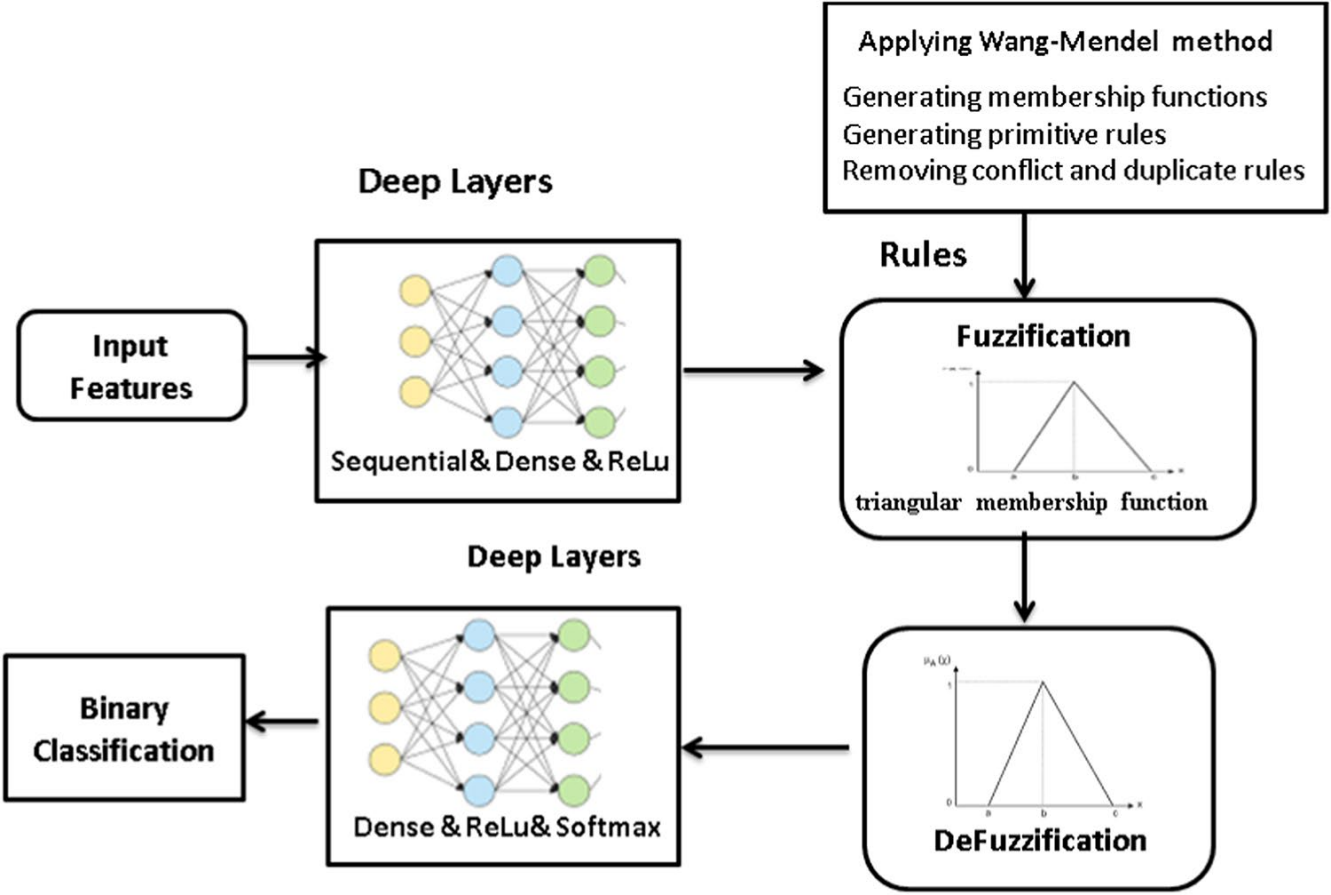
Structural design of fuzzy deep classifier

The fuzzy architecture is made up of four basic components which are Fuzzification, fuzzy membership function, inference engine, and Defuzzification as shown in Fig. 5.

#### Fuzzy membership function

The membership function, which determines the scope to which an input value belongs to a particular group, is the essential idea behind fuzzy logic. In this paper, triangular membership function is applied because it is simple to implement and fast for computation. It can be calculated as the following:

10 $${\text{Triangular}} = \left\{ {\begin{array}{*{20}l} 0 \hfill & {x \le a} \hfill \\ {\frac{{x - a}}{{b - a}}} \hfill & {a \le x \le b} \hfill \\ {\frac{{c - x}}{{c - b}}} \hfill & {b \le x \le c} \hfill \\ 0 \hfill & {c \le x} \hfill \\ \end{array} } \right.$$

where $c$ describes the base and $b$ states the height of the triangle.

#### Fuzzification layer

The Fuzzification layer called the first layer that selects the membership functions that correspond to the input values. In the Fuzzification layer, the crisp data transformed into fuzzy data sets by applied membership functions using the following equation:

11 $${\alpha }_{is}=\prod_{j=1}^{N}{\mu }_{ij}({x}_{sj})$$

Where $N$ is the features numbers and ${\mu }_{ij}$ is the membership function

#### Defuzzification layer

Defuzzification is the process of reducing an aggregated fuzzy set’s output to a single number that is used to determine a certain class. Defuzzification transforms fuzzy values crisp values using the following equation:

12 $${\beta }_{sk}=\sum_{i=1}^{M}{\alpha }_{is}{w}_{ik}$$

#### Generating fuzzy rules

Fuzzy rules are generated by algorithm 4 that using the feature selected from algorithm 3. The algorithm first determines the per ntage of zero values in each column to discover and remove columns that have more than 10% zeros. It then calculates the correlation matrix between the target variable $y$ and the other features. The most effective features are chosen and collected based on their strongest connect to y. After concatenating these features with y, a new dataset is created and shuffled to make sure randomization. After that, the Wang-Mendel approach is used, which involves creating basic fuzzy rules, producing membership functions for the features and y, and clustering the data into fuzzy clusters. To preserve consistency, any contradictory or redundant rules are eliminated. Finally, the improved set of fuzzy rules is produced and prepared for use in the identification of cancer.

**Algorithm 4:**
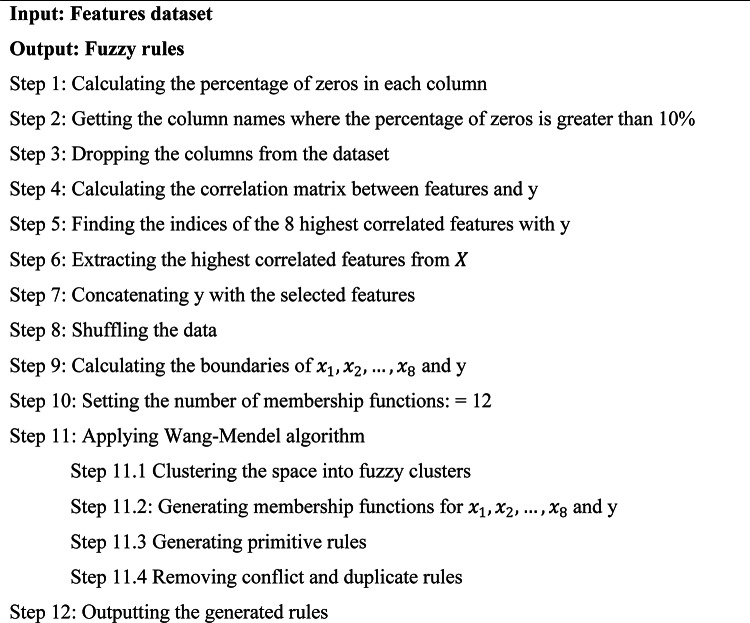
Generating fuzzy rules

#### Deep learning layers

There is an activation function between each layer of neurons. This layer transforms the output of one that is fed into the next, with varying effects on the network’s ability to detect the issue. The most frequent activation function is known as ReLU and SoftMax. The neural network’s raw outputs are transformed into a vector of probabilities by the softmax activation function, which also makes sure that each output is precisely equivalent to 1. The probability of a data point belonging to each individual class is provided by the SoftMax activation function using the following equation.

13 $$\sigma {(\overrightarrow{z)}}_{i}=\frac{{e}^{{z}_{i}}}{\sum_{j=1}^{k}{e}^{{z}_{j}}}$$

Where $\overrightarrow{{\varvec{z}}}$ is the input vector, ${{\varvec{e}}}^{{{\varvec{z}}}_{{\varvec{i}}}}$ is the exponential function for input vector, $K$ is the number of classes, ${{\varvec{e}}}^{{{\varvec{z}}}_{{\varvec{j}}}}$ is the exponential function for output vector. The use of ReLU helps to stop the neural network’s computation from growing at an exponential rate. Additionally, ReLUs also stop the vanishing gradient problem from happening. ReLU layer is an activation layer and it returns 0 if it takes any negative input; However, the function returns the positive value it receives for x. It can be computed using the following equation:

14 $$f\left(x\right)=\left\{\begin{array}{c}0 for x<0\\ x for x\ge 0\end{array}\right.$$

Algorithm 5 applied the fuzzy deep learning classifier that can distinguish between cancer and non-cancer responses by using the best features as input. Initially a dense input layer representing the best-selected features for the classification task is created, with a size of 10,000. The ReLU is then applied to this input layer to give the model non-linearity and preserving computing efficiency. Through a fuzzification layer, the algorithm uses the membership functions to convert the crisp input data into fuzzy data sets. To execute this conversion, the total fuzzy membership value for every fuzzy rule is calculated. The model adds a dense hidden layer after fuzzification to process the modified fuzzy inputs even further. The ability of the model to identify complex patterns and interactions within the data is improved by this hidden layer. This hidden layer is subjected to another application of the ReLU activation function, which strengthens the non-linear transformation of the data and enhances the model’s overall learning capacity.

In summary, the model can effectively handle complicated and confusing data patterns through the application of fuzzy logic and deep learning techniques.

**Algorithm 5:**
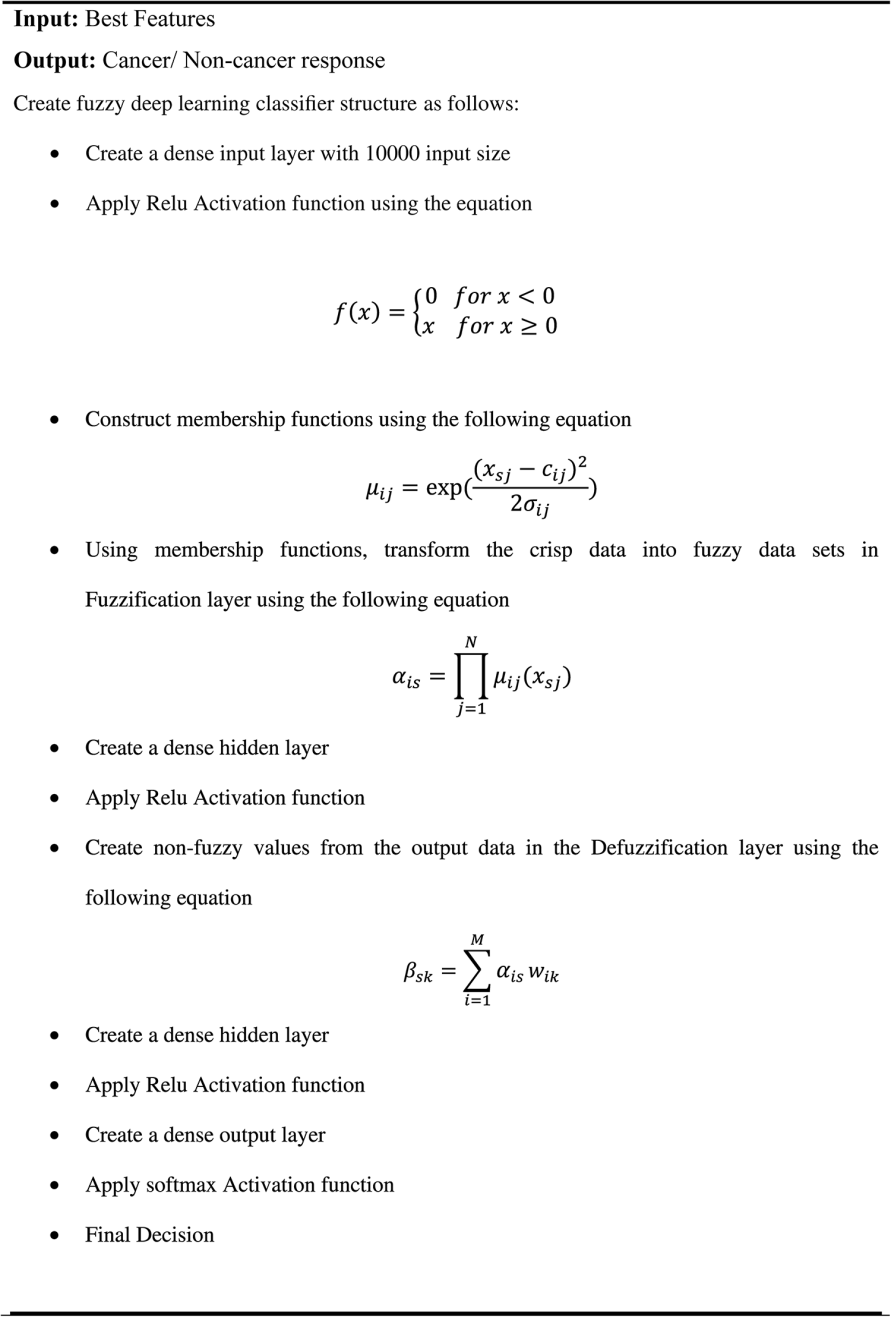
Fuzzy deep learning classifier

## Experimental results and discussion

The main components of the implementation environment, experiment setup, parameter initialization, and performance measurements are discussed in the following subsections.

### Experimental parameters

All software codes are written in Python 3.6.13. Experiments run on an HP Envy laptop with a seventh Generation AMD Rayzen, 16 GB RAM, 64-bit Windows 11 Operating System. Famous machine learning modules employed in the field of artificial intelligence are TensorFlow and Keras. Compared to other top platforms, Tensor Flow offers a superior graph representation for a given set of data. One benefit of Tensor Flow is that it supports and makes use of a variety of backend programmes, such as GUIs. TensorFlow platform’s high-level API is called Keras. With an emphasis on contemporary deep learning, it offers an easy-to-use, extremely productive interface for resolving machine learning (ML) issues. Every stage of the machine learning process is covered by Keras, including data processing, hyperparameter tuning, and deployment. Its development was centred on making quick experiments possible. In this model, data is split with ratio 80% into train and 20% test subsets

### Performance metrics

A variety of metrics are used to assess the performance of our model, using recall, F-measure, accuracy, and precision. The metrics’ definitions are as follows:

15 $$precision=\frac{TP}{TP+FP}$$

16 $$Recall=TPR=\frac{TP}{TP+FN}$$

17 $$\text{F}1-\text{Score }=\frac{2TP}{2TP+FP+FN}$$

18 $$\text{Accuracy}=\frac{TP+TN}{TP+TN+FN+FP}$$

19 $$Specificity = TNR=\frac{TN}{TN+FP}$$

Where $FP, FN, TP and TN$ is the False Positive, False Negative, True Positive, and True Negative, respectively.

### System Walkthrough

**Fig. 6:**
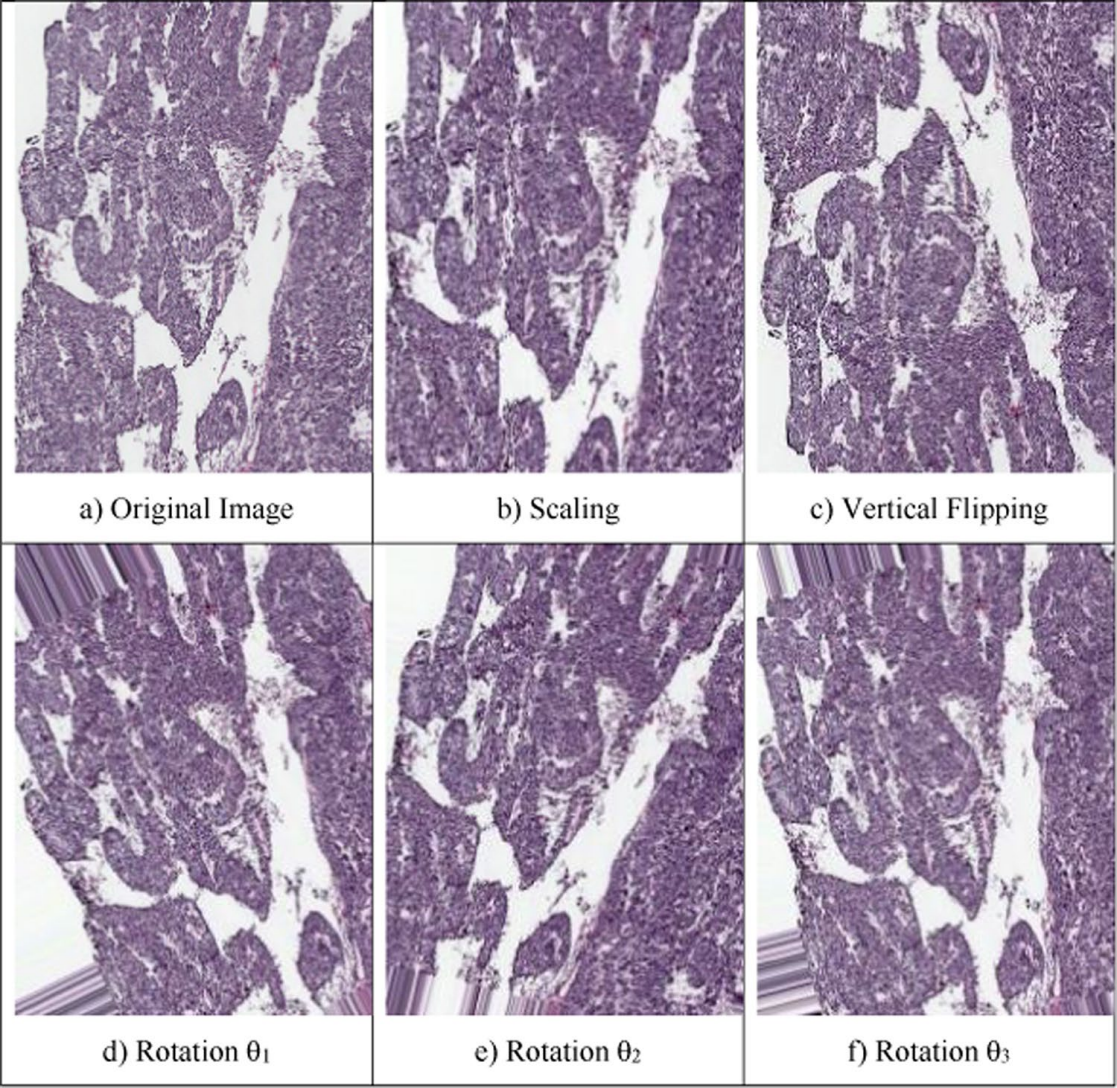
Image augmentation results

As depicted in Fig. 6, the images in the dataset undergo augmentation such as scaling with scaling factor 20%, vertical flipping, and 3 rotations within the range of 50°.

Table 2 shows the number of each class before and after augmentation. In our dataset, there are 506 non-cancer samples without augmentation and 481 samples in the cancer class. After augmentation, there are 2,405 for cancer class and 2530 for non-cancer class.

**Table 2:** Size of the dataset before and after augmentation

Dataset	Before augmentation	After Augmentation				
		Scaling	Vertical	Rotate θ_1_	Rotate θ_2_	Rotate θ_3_
No. of Cancer	481	481	481	481	481	481
No. of Non-cancer	506	506	506	506	506	506

The weights of the network may be iteratively updated during training data using the optimization algorithm Adam, which can be employed in place of the conventional techniques. Different parameters are used to train our model, as indicated in Table 3. The proportion at which weights are updated is indicated by the learning rate, also known as alpha, which is equal to 0.001. The batch size, which is 32 samples, determines how many samples we use to train a neural network in one epoch.

**Table 3:** Adam value optimizer: hyperparameter setting

Parameter	Value
Optimizer	adam
Learning rate	0.001
Loss function	Cross entropy
Batch size	32
Epochs	100

**Fig. 7:**
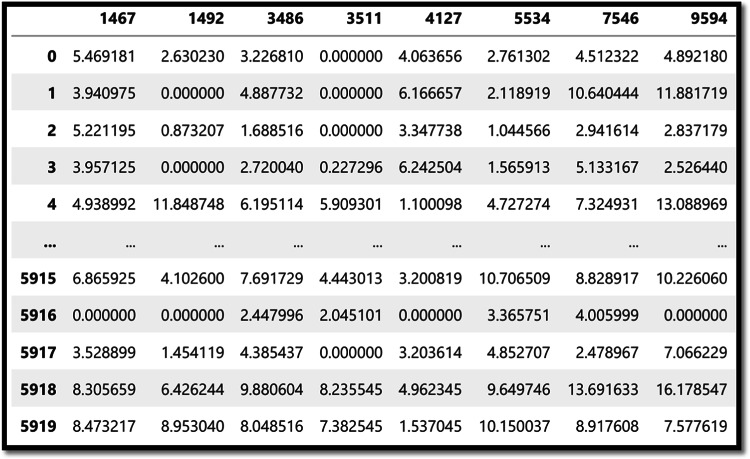
Eight highest correlated features

Figure 7 shows the eight highest correlated features with the target, ensuring that no column in the dataset has more than 50% of its values set to zero.

**Fig. 8:**
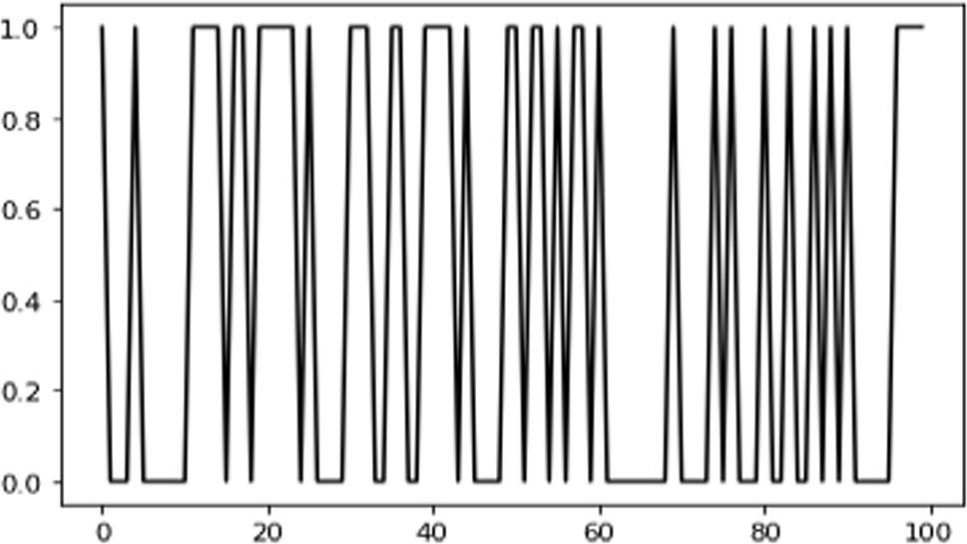
First 100 components of Y_train_ data

**Fig. 9:**
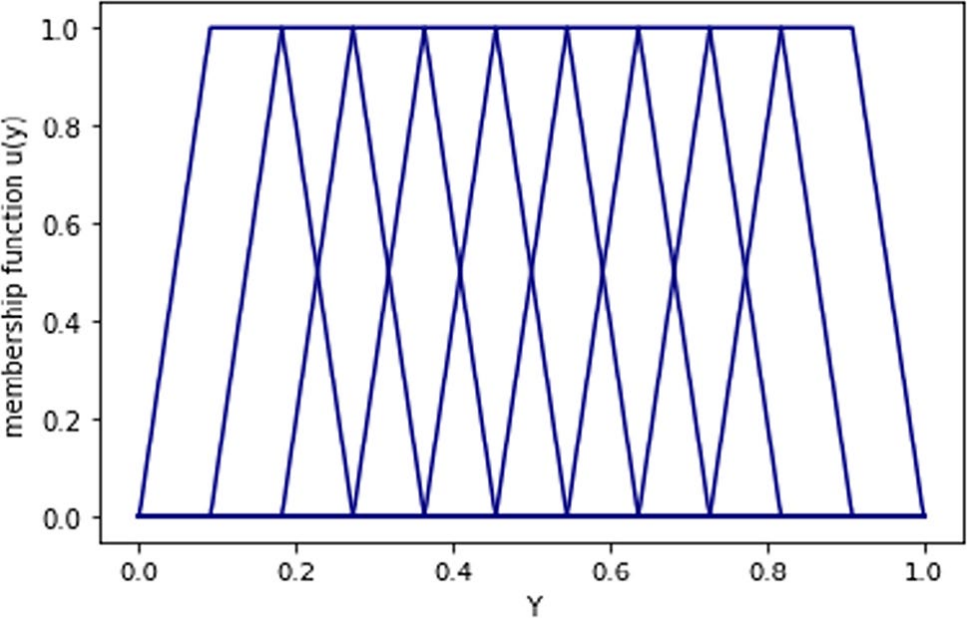
Membership functions of y

Figure 8 shows the first 100 components of the target variable values used in training (Y_train_) while Fig. 9 shows the membership function of Y.

There are 5000 primitive rules. Then there are 5000 non-conflicting and non-duplicated rules for each class response. Table 4 shows a sample of the generated rules. For the rule R_0_: If x1 is 2 and x2 is 7 and x3 is 4 and x4 is 8 and x5 is 0 and x6 is 2 and x7 is 3 and x8 is 4 then y is 0.

**Table 4:** Generated set of rules

id	x1	x2	x3	x4	x5	x6	x7	x8	y
0	2	7	4	8	0	2	3	4	0
1	2	6	2	8	0	2	1	1	0
2	2	6	3	7	1	2	2	2	0
…									
5000	0	20	2	4	19	16	7	16	1
5001	7	6	20	19	8	15	20	15	1
5002	2	7	3	6	11	18	17	7	1
…									

**Fig. 10:**
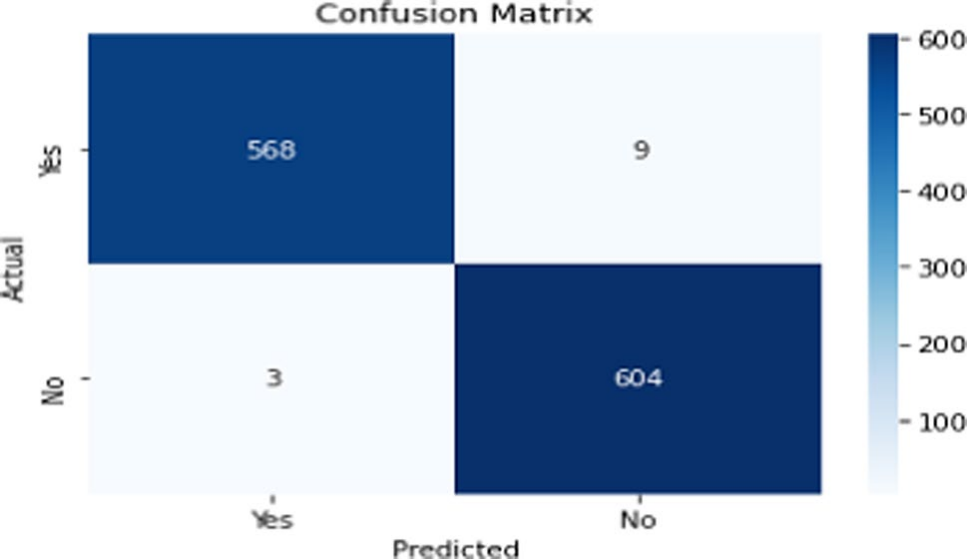
Confusion Matrix

**Fig. 11:**
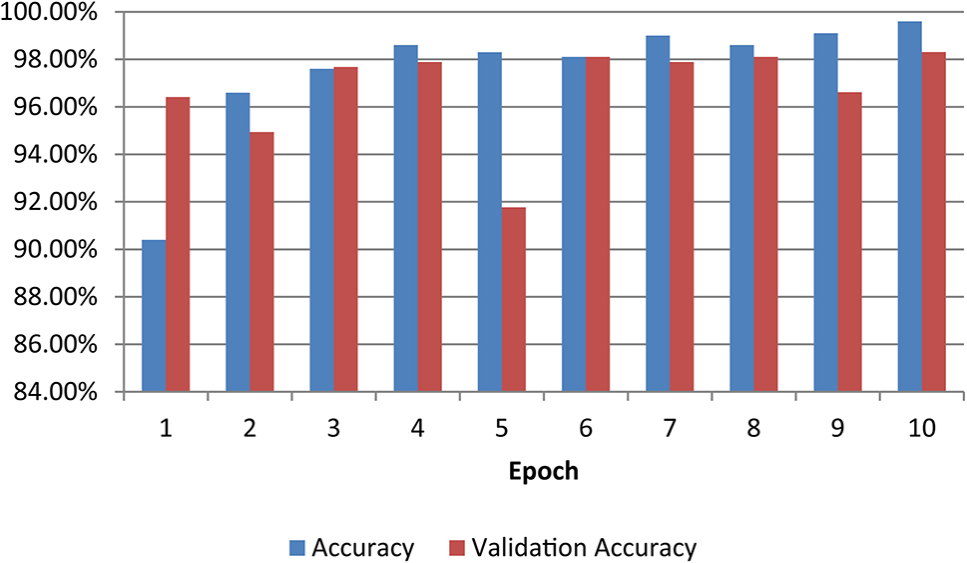
Accuracy and validation accuracy on different epoch

The confusion matrix for the proposed model is shown in Fig. 10. It demonstrates how the proposed model correctly identified the images of ovarian cancer. In addition, Fig. 11 shows the accuracy and validation accuracy from 1 to 10 epochs.

Several metrics are used to evaluate the performance of the suggested model, including recall, F-measure, accuracy, and precision as shown in Table 5. The average results achieved 99.99% accuracy, 99% sensitivity, 98.96% specificity, and 98.99% F1-score.

**Table 5:** Performance metrics of the classifier

Metric	Cancer class	Non-cancer class	Average
Accuracy	99%	98.97%	98.99%.
Precision	99.47%	98.53%	99%
Recall	98.44%	99.51%	98.96%
F1-score	98.95%	99.02%	98.99%

A comparison of three different membership functions known as Trapezoidal Piecewise Linear Triangular is shown in Table 6. During the training model, Generation Time (s), Train Error, and Test Error are calculated. A training error is just an error that happens when the model is being trained; for example, a dataset may be handled incorrectly during preprocessing or feature selection. However, testing errors varies slightly, such as underfitting and overfitting of the model. The triangle membership function produced superior results and reduced training time, as this table illustrates.

**Table 6:** A comparison of membership functions

Membership function	Training time (s)	Train error	Test error
Trapezoidal	0.0191305	0.1949	0.1957
Piecewise linear	0.0231216	0.1954	**0.1933**
Triangular	**0.0116997**	**0.1947**	0.1979

Significant values are in [bold]

Table 7 compares the accuracy of various models. The model achieved 98.07% accuracy when it employed Deep Learning for classification and ResNet50 for feature extraction. The next model uses ResNet50 for feature extraction, applies RFE for feature selection, and uses Fuzzy Deep Learning for classification. With 10,000 features, this model achieved a 98.99% accuracy. The last model applied RFE for feature selection, ResNet50 for feature extraction, and Light Gradient-Boosting Machine for classification. The accuracy of this model is 98.92% when there are 10,000 features.

**Table 7:** Accuracy comparison for of models

Model	No. of features	Accuracy (%)
ResNet50 & Deep learning	100,352	98.07
ResNet50 & RFE & Fuzzy deep learning	10,000	98.99
ResNet50 & RFE &LGBM	10,000	98.92

**Fig. 12:**
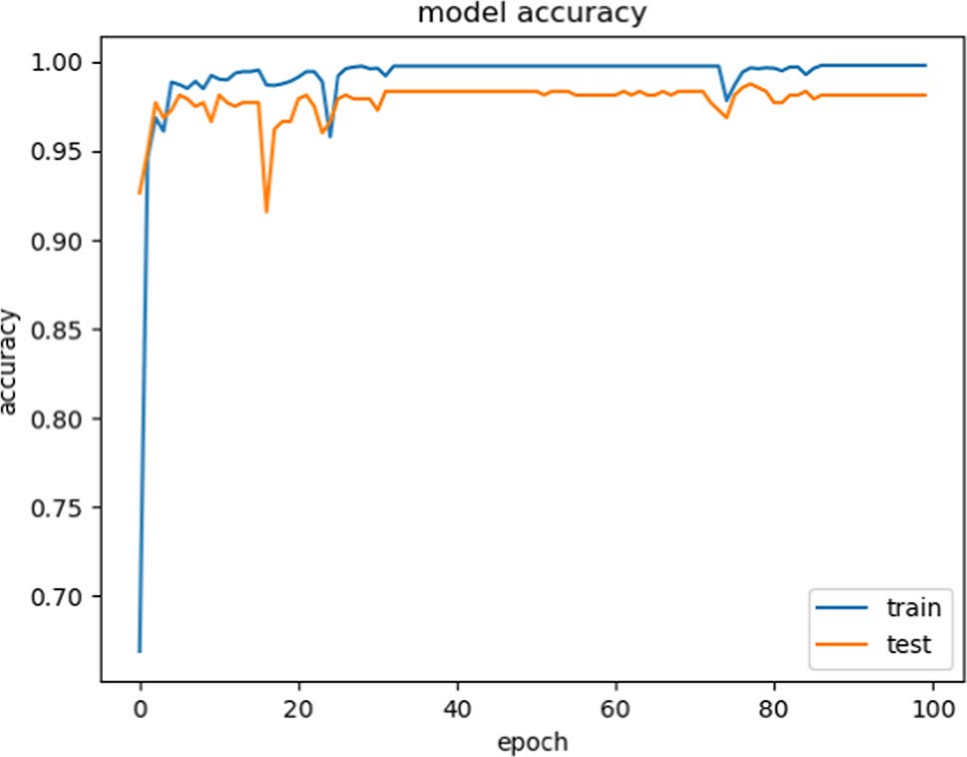
Model Accuracy: Training vs. Testing

**Fig. 13:**
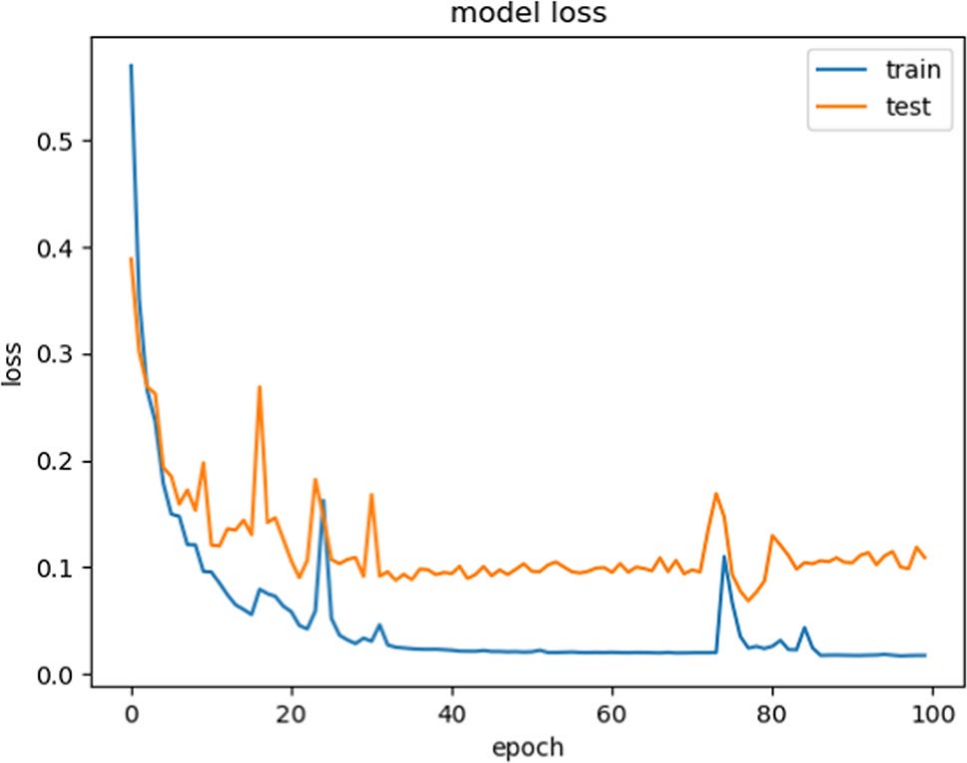
Model Loss

Accuracy does not necessarily increase with epoch size. In a neural network, all of the training data had been utilized for optimizing the model parameters after one epoch. The perfect epochs are between 1 and 100. Figure 12 shows the epoch versus accuracy. Accuracy rises with the number of epochs before being saturated. While the epoch versus loss of the model on train and validation datasets is shown in Fig. 13.

The accuracy of the proposed model’s results and the state-of-the art is shown in Table 8. Two experiments were done by the authors in^17^. The dataset for the first experiment is divided into two separate subsets (65% training and 35% testing) and 5-fold cross validation is carried out for the second experiment. For each experiment, three techniques are performed. It has been found that the proposed model is more accurate than the other models.

**Table 8:** Comparing the proposed model’s accuracy and previous work.

Ref.	Techniques	Accuracy
65% training, 35% testing^17^	Cascaded deep learning	76.2%
	Decision voting model	85.1%
	Data augmentation	82.2%
5-fold cross validation^17^	Cascaded deep learning	82.2%
	Decision voting model	87.1%
	Data augmentation	88.2%
Proposed model	ResNet-50 and fuzzy deep classifier	98.99%

## Conclusions and future work

This paper provides an accurate model for classifying ovarian cancer using a fuzzy deep learning algorithm. The dataset consists of 288 complete hematoxylin and eosin (H&E)-stained slides with clinical information from 78 patients. Various tissue blocks of post-treatment specimens were used to create H&E-stained Whole Slide Images (WSIs); of these, 162 valid and 126 invalid WSIs were obtained. The model is composed of the crucial four parts which are: augmentation, feature extraction, feature selection, and finally classification. Initially, rotation, scaling, and vertical filliping are used to increase the dataset. The ResNet50 model, a simple CNN with 50 deep layers, is then used to extract the features from the histopathological images. Recursive feature elimination is a vital technique for increasing the effectiveness of classification of images by removing pointless or unimportant elements. Finally, a benchmark dataset is trained and classified using a fuzzy deep learning classifier. The proposed model in this paper has a promising accuracy of up to 98.99% for distinguishing between images of ovarian cancer and Non-cancer. Compared to similar models, it demonstrated promising accuracy and shown higher performance. Future research in this field might involve extending the dataset to diagnose other varieties of precision oncology and increasing the classification accuracy of the algorithm.

## Data Availability

The data has been accepted for publication on The Cancer Imaging Archive (TCIA), and the dataset presented is available at 10.7937/tcia.985g-ey35

## Code availability

The code that supports the findings of this paper is available from author Mohamed El-dosuky, upon reasonable request.
